# Understanding the Interplay of Maternal Mental Health, Social Support, and Sociodemographic Factors in Promoting Exclusive Breastfeeding in Kinshasa

**DOI:** 10.3390/nu18010065

**Published:** 2025-12-25

**Authors:** Gloria B. Bukasa, Francis K. Kabasubabo, Berthold Matondo Bondo, Din-Ar B. Batuli, Pierre Z. Akilimali

**Affiliations:** 1 Renaissance University Hospital Centre, Health Zone of Gombe, Kinshasa P.O. Box 11850, Democratic Republic of the Congo; globukasa@gmail.com; 2 Patrick Kayembe Research Center, Kinshasa School of Public Health, University of Kinshasa, Kinshasa P.O. Box 11850, Democratic Republic of the Congo; fkabasu13@gmail.com; 3 Inserm U1094, IRD UMR270, CHU Limoges, EpiMaCT-Epidemiology of Chronic Diseases in Tropical Zone, Institute of Epidemiology and Tropical Neurology, OmegaHealth, University of Limoges, 87000 Limoges, France; 4 Mère et Enfant Barumbu General Referral Hospital, Health Zone of Kinshasa, Kinshasa P.O. Box 11850, Democratic Republic of the Congo; bmbondo900@gmail.com; 5 General Referral Hospital of Makala, Health Zone of Selembao, Kinshasa P.O. Box 11850, Democratic Republic of the Congo; btlbopete@gmail.com; 6 Department of Nutrition, Kinshasa School of Public Health, University of Kinshasa, Kinshasa P.O. Box 11850, Democratic Republic of the Congo

**Keywords:** breastfeeding self-efficacy, exclusive breastfeeding, dietary diversity, Kinshasa

## Abstract

Background: Exclusive breastfeeding (EBF) is crucial for infant health, and maternal mental health significantly influences breastfeeding practices. This study investigates the relationships among postpartum depression (PPD), maternal dietary diversity, and exclusive breastfeeding in Kinshasa, Democratic Republic of Congo. Methods: A cross-sectional study was conducted involving 793 mother–child pairs. Data were collected through structured interviews using a validated questionnaire administered by trained enumerators. Statistical analyses included descriptive statistics, chi-square tests, and structural equation modeling (SEM) to evaluate the relationships between maternal and child characteristics and EBF. Results: The proportion of infants in the study sample who were exclusively breastfed was 29.1% (95% CI: 26.0–32.3%). Breastfeeding self-efficacy is positively associated by nutritional advice during pregnancy, with a coefficient of 2.17 (*p* = 0.003). The husband’s support in exclusive breastfeeding positively correlates with breastfeeding self-efficacy (coefficient = 0.23, *p* < 0.001). A significant negative relationship exists between child age and EBF (coefficient = −0.095, *p* < 0.001). EBF is positively associated by nutritional advice during pregnancy, with a coefficient of 0.12 (*p* = 0.016). Child morbidity in the last 2 weeks showed a negative association with EBF practice (coefficient = −0.09, *p* = 0.014). Conclusions: This study highlights the multifaceted challenges faced by mothers in Kinshasa regarding exclusive breastfeeding. By prioritizing husband involvement, nutritional counseling, and robust health-system engagement, we can create a more supportive framework for breastfeeding practices. Future research should focus on longitudinal approaches to understand the long-term impacts of these factors on breastfeeding and infant health. Additionally, exploring the potential benefits of integrated maternal health programs that address nutritional needs will be crucial in developing comprehensive support systems for new mothers.

## 1. Introduction

Exclusive breastfeeding (EBF) is universally acknowledged as a vital strategy for enhancing infant survival and development. The World Health Organization (WHO) defines EBF as the practice of feeding an infant solely with breast milk without water or other solid or liquid foods, except for syrups and vitamins, during the first six months of life [1,2]. This practice plays a crucial role in reducing both infant morbidity and mortality, as it safeguards against infections and promotes optimal nutritional and cognitive development [3,4]. Infants who are exclusively breastfed for the first six months of life are 14 times less likely to die from causes such as pneumonia and diarrhea compared to non-breastfed infants [3]. Despite the well-documented advantages of EBF, child malnutrition persists as a significant public health challenge in the Democratic Republic of Congo (DRC). Alarmingly, the latest Demographic and Health Survey (EDS-III, 2023–2024) reveals that 45% of children under five in the DRC are affected by stunted growth, while 7% face acute malnutrition [5].

Understanding the factors contributing to these high rates of malnutrition is essential, especially since breastfeeding alone may not address all underlying issues. Breast milk is a complete source of nutrition essential for infant growth and development, providing all necessary nutrients in optimal proportions [6]. It offers significant immunological protection, supporting infants’ health by enhancing their immune response and promoting gut health. This dynamic composition adapts over time to meet the changing needs of the growing infant, making breastfeeding vital for both immediate and long-term health outcomes [3,6].

However, current EBF rates in the DRC are low, with only 53% of infants under six months being exclusively breastfed, and this figure drops to 25% in Kinshasa [5]. Several socioeconomic factors, cultural norms, and maternal health conditions impact these rates, particularly postpartum depression (PPD) [7], which is associated with 10% to 30% of women in sub-Saharan Africa. PPD associated with symptoms that can reduce mothers’ motivation and ability to breastfeed, often resulting in premature weaning and increased reliance on substitutes, posing risks to infant health [8,9]. PPD also disrupts the hormonal balance necessary for milk production, potentially decreasing supply. Effective support systems, including mental health resources and educational initiatives, are vital in helping mothers manage PPD and encouraging breastfeeding, benefiting both maternal health and infant nutrition [10]. Furthermore, PPD can severely hinder a mother’s ability to initiate and maintain EBF [10,11,12]. Understanding these interconnected aspects is crucial for designing effective interventions to promote and sustain breastfeeding practices throughout Africa.

In recent years, Africa has witnessed a concerning decline in exclusive breastfeeding rates. This downturn poses serious implications for newborn health and nutrition in a region where breastfeeding is critical for enhancing child survival and well-being. This decline can be attributed to various factors, including the aggressive marketing of complementary foods, formula milk, and cereals, that undermine breastfeeding practices [13,14]. Additionally, the interplay between a mother’s self-efficacy in breastfeeding and the level of support from male partners significantly influences breastfeeding outcomes.

Data from the Demographic and Health Surveys (2007, 2013, and 2023) and Multiple Indicator Cluster Surveys (2010 and 2017) [5,15,16,17,18] reveal a troubling trend: while the national landscape in the DRC shows signs of improvement, Kinshasa is experiencing a dramatic decline in exclusive breastfeeding rates. As of 2023, only 25.3% of infants in Kinshasa are exclusively breastfed, starkly lower than the national average of 52.3% [5]. Immediate action is imperative to identify and address the factors contributing to this reality in Kinshasa, thereby promoting breastfeeding as a public health priority. Previous studies, such as those conducted by Wallenborn et al. [19], have indicated significant disparities in breastfeeding practices between urban and rural settings. Factors such as urban lifestyle changes, cultural shifts, media influence, lack of familial support, and peer pressure are believed to play a role in the declining rates of EBF in Kinshasa [20]. Urban areas also tend to exhibit higher incidences of unplanned pregnancies than rural regions [21]. Moreover, mental health issues critically affect a mother’s ability to initiate and sustain breastfeeding [10,22].

The aim of this study was to investigate the interplay between PPD, EBF, and associated factors such as self-efficacy, social support, dietary diversity, and child morbidity within a unified structural model. Despite some research linking intended birth to higher rates of PPD, there is a lack of comprehensive studies examining how these variables collectively influence breastfeeding practices in the DRC [23,24]. By employing structural equation modeling (SEM), we sought to identify psychological barriers to EBF and inform targeted interventions that address the multifaceted nature of maternal and child health. Ultimately, we aim to guide healthcare practices and policies that promote EBF, considering the diverse influences on maternal decisions and experiences, thereby enhancing health outcomes for mothers and infants.

## 2. Methods

### 2.1. Study Design and Participants

A cross-sectional analytical design was employed in this study. The research was conducted from 15 September to 8 October 2025 in seven main hospitals in Kinshasa City (CME BARUMBU, CS MOSOSO, General Reference Hospital of MATETE, Maternity DE BINZA, Maternity de Kingasani, Maternity of Kintambo, and Hospital Saint Gabriel). The maternity wards in Kinshasa have the highest volume of prenatal, preschool, and postnatal consultations, serving all socioeconomic demographics within the city province. The target population consisted of mother–child dyads, specifically mothers aged ≥18 years with children aged 0 to 6 months recruited during preschool and postnatal consultations across the above health facilities. This child age group was selected due to its critical importance for breastfeeding practices and its influence on child growth and development. Exclusions were made for children with severe pathologies and mothers experiencing significant obstetric complications.

### 2.2. Sample Selection

The sample size was determined using the formula for estimating proportions in cross-sectional studies (OpenEpi), taking into account a 95% confidence level and an estimated exclusive breastfeeding (EBF) prevalence of 53% (EDS-III/RDC, 2023–2024) [5]. An anticipated minimum required sample of 766 mother–child pairs was adequate using a clustering effect of 2. Considering a non-response rate of 5%, the sample size was increased to 806. All mothers of children aged 0 to 6 months attending the seven above maternities for preschool and postnatal consultations during the data collection period were invited to participate. We employed a consecutive-based sampling method, ensuring that all eligible mother–child pairs during the recruitment period were included. The final number of participants was reduced from 806 to 793 (response rate of 98.4%).

### 2.3. Variables of the Study

The primary dependent variable was the practice of exclusive breastfeeding (EBF), defined by the WHO as feeding a child aged 0 to 6 months solely with breast milk, without any water, other liquids, or solid foods. EBF was measured through direct interviews with mothers using a 24 h dietary recall, expressed both as a binary variable and as a percentage of children exclusively breastfed the day prior to the survey. To compare estimated measures of exclusive breastfeeding, we employed three methods based on large-scale household surveys:

1. EBF-24H: Prevalence of exclusive breastfeeding among infants aged less than 6 months based on a 24 h recall.
2. Self-Declaration: EBF status based on a one-question self-declaration by the mother: “Did you feed your child only breast milk yesterday?”
3. EBF-SB: Percentage of infants aged less than 6 months who had not been given anything other than breast milk since birth.

The secondary dependent variable was the Breastfeeding Self-Efficacy Scale—Short Form (BSES-SF). This self-report questionnaire evaluates the self-efficacy levels of breastfeeding mothers. The BSES-SF consists of 14 items rated on a five-point Likert scale, with higher scores indicating greater breastfeeding self-efficacy [25]. The internal consistency of the scale was assessed, and a Cronbach’s alpha of 0.87 was calculated in this study.

The main independent variables were dietary diversity, the Edinburgh Postnatal Depression Scale (EPDS), the husband’s (or male partner’s) postpartum social support, and child morbidity. Dietary intake was assessed using a 24 h recall method. Foods were classified into the ten Minimum Dietary Diversity for Women (MDD_W) food groups [26]. A point was allocated for each group consumed, with a range of 0 to 10. Adequate dietary diversity is defined as MDD_W ≥ 5, while inadequate diversity is indicated by MDD_W < 5, serving as a binary indicator. The cumulative MDD-W scores were categorized into variables: adequate dietary diversity (consuming five or more food groups) and inadequate dietary diversity or dietary monotony (consuming fewer than five food groups) [26]. Dietary diversity was measured according to a single 24 h recall using the MDD_W food group classification. We acknowledge that a single day may not reflect habitual intake, increasing the possibility of random measurement error; this limitation was anticipated and is discussed below. The Edinburgh Postnatal Depression Scale (EPDS) [27] is an internationally validated scale comprising 10 items reflecting symptoms experienced in the past week, such as sadness, anxiety, and feelings of incompetence. The total score can be anywhere from 0 to 30, and each item is rated from 0 to 3 based on the severity of the symptoms. A score of ≥13 indicates the presence of probable PPD, allowing the identification of mothers potentially experiencing psychological distress that may influence EBF practices. The internal consistency of the scale was assessed, and a Cronbach’s alpha of 0.84 was calculated in this study. The husband’s (or male partner’s) support in exclusive breastfeeding tool included seven items assessing spousal support using a five-point Likert scale (see Table S1). The items were derived from consultations with specialists in maternal and child health, as well as prior studies executed in Kinshasa [28,29,30,31]. These items were formulated in conjunction with lactation experts. This questionnaire was evaluated with a target group to ascertain its clarity and relevance, hence enhancing the validity of the questions. The internal consistency of the scale was assessed, and a Cronbach’s alpha of 0.76 was calculated in this study. To accurately assess the incidence of illness (morbidity in the last 2 weeks) among children in the two weeks leading up to the survey, the child was considered to have had an illness when they encountered at least one of three childhood illnesses (acute respiratory infections/cough, diarrhea, fever) and categorized as “yes”, while those who had none of them were categorized as “no” [32]. This approach was implemented to mitigate information bias and ensure reliable data collection. One in four observations missed this data.

The other covariates included basic characteristics (ages of participant and child, mother’s educational level, religion, marital status, number of prenatal consultations attended, parity, household size, complications during pregnancy or childbirth, nutritional advice during pregnancy, child sex, and childbirth weight). The DHS Equitool was employed for household respondent wealth classification [33].

### 2.4. Data Collection

The data collection process was carefully crafted to guarantee high-quality results through structured individual interviews using a validated questionnaire. A comprehensive training program was implemented to ensure inter-rater reliability among enumerators, incorporating standardized protocols and clear instructions on data gathering methodologies. After the training, enumerators participated in a series of evaluations to check their consistency and accuracy in reporting, facilitating modifications and recommendations to improve dependability prior to the initiation of data collection. The data collection tools were pre-tested and checked for consistency then modified as deemed necessary. Data was gathered utilizing smartphones configured with SurveyCTO version 2.81.3. After data collection, the information was securely stored on a dedicated server, followed by a quality examination before analysis in Stata 18 (StataCorp, College Station, TX, USA). A team of 15 trained enumerators undertook the task and were supervised daily for consistency and accuracy over a 21-day period. Daily consistency checks identified discrepancies, ensuring reliability. Interviews were conducted in Lingala, the vernacular language in Kinshasa, or French. In consultation with bilingual researchers, we used backward translation to translate items between French and Lingala to ensure linguistic and conceptual equivalence. Entries failing predetermined validity criteria, such as incomplete responses, were excluded to ensure that only high-quality data contributed to the findings. This meticulous approach was employed to preserve data integrity and enhance the validity of the results. During the initial phase of data collection, we discovered that the ODK programming was unable to capture morbidity information; consequently, we revised the ODK form to include this critical data, ensuring a more comprehensive understanding of participant health.

### 2.5. Statistical Analysis

Descriptive statistics were calculated for all variables. Proportional estimates and confidence intervals for EBF status were derived, and chi-square tests were conducted to assess relationships among categorical variables. *T*-tests were employed to compare means of continuous variables across EBF categories. A Venn diagram was utilized in the analysis to illustrate the interrelations among three principal variables concerning exclusive breastfeeding: self-reported exclusive breastfeeding (self-declaration), exclusive breastfeeding at 24 h (EBF-24H), and exclusive breastfeeding as determined according to EBF-SB. A total of 793 records were examined, uncovering significant insights into the intersection and distribution of these variables.

Proportional estimates and confidence intervals for EBF status were derived, and chi-square tests were conducted to assess relationships among categorical variables. *T*-tests were employed to compare means of continuous variables across EBF categories. Logistic regression was used to evaluate the associations of independent variables with EBF status, adjusting for potential confounders.

Structural equation modeling (SEM) was employed in this study to explore the relationships between various factors influencing exclusive breastfeeding practices among a sample of 793 participants. Initially, we conducted bivariate analyses to examine the relationships between all independent variables and the two dependent variables: the Breastfeeding Self-Efficacy Scale and exclusive breastfeeding in the last 24 h. This initial analysis was crucial for identifying significant associated factors that informed the SEM pathways, as supported by the foundational literature on structural equation modeling [34,35,36]. The significant relationships identified in the bivariate analyses guided the SEM, which allows for the assessment of latent variables and testing of hypothesized relationships at the theoretical level [37]. Thus, SEM was the preferred analytical strategy for simultaneously analyzing the constructs of maternal characteristics, infant characteristics, postnatal complications, breastfeeding techniques, and exclusive breastfeeding initiation within our hypothetical model [38].

The SEM analysis utilized maximum likelihood estimation with robust standard errors, accounting for clustering within facilities. The model fit was evaluated through iterations of log pseudolikelihood and goodness-of-fit statistics, ensuring the robustness of relationships among variables. Endogenous variables included the Breastfeeding Self-Efficacy Scale and exclusive breastfeeding in the last 24 h, while exogenous variables comprised the child age, religion, dietary diversity score, nutritional education, child’s morbidity in the last two weeks, husband’s support for exclusive breastfeeding, probable major maternal depression, mother’s educational attainment, mode of birth, complications during pregnancy, and socioeconomic status. This comprehensive methodology enabled a clearer understanding of the factors contributing to breastfeeding practices and set the stage for targeted interventions.

Missing data was handled using multiple imputations by chained equations with m = 20 imputations. We combined estimates using Rubin’s rules and contrasted results with a complete-case analysis (Table S2). The imputation model included all variables from the analytical model plus auxiliary predictors related to missingness. Convergence and plausibility were assessed via trace and over-imputation diagnostics. In the sensitivity analysis, we ran SEM with the default standard error, and the strength of fit of the SEM model was investigated based on multiple indices, including the root-mean-square error of approximation (RMSEA), comparative fit index (CFI), and Tucker–Lewis’s index (TLI). The following cut-off values, which were suggested by Hu and Bentler [39], indicate a good fit: our RMSEA  was 0.011, which is less than  0.06; the coefficient of determination was 0.235; and the CFI and TLI  were 0.987 and 0.963, respectively, which are over 0.95. The significance level of *p* < 0.05 was set to ensure robust results.

### 2.6. Ethical Considerations

The protocol was approved by the Ethics Committee of the School of Public Health of Kinshasa (approval no. ESP/CE/83/2025, on 11 April 2025). Informed consent was obtained from each participant after explaining the study objectives and their right to refuse or withdraw without consequences. Confidentiality was maintained by anonymizing data and storing it securely on a protected computer accessible only to the research team.

## 3. Results

The findings from this study on exclusive breastfeeding practices involving 793 mother–child pairs are presented, focusing on themes such as participant characteristics, dietary diversity, maternal mental health, and breastfeeding self-efficacy. The detailed statistical analyses, supported by tables and figures, highlight the relationships between sociodemographic factors, nutritional advice, and breastfeeding outcomes. These insights are essential for understanding the complexities of exclusive breastfeeding in the study population and are intended to guide future interventions for enhancing maternal and infant health.

### 3.1. Participant Characteristics

The sociodemographic characteristics of the participants are summarized in Table 1. The average age of mothers participating in the study was approximately 29 years, with a significant majority (about 58.8%) falling within the age range of 25 to 34 years. Most mothers (93.1%) had completed secondary education or higher, and 87.3% reported being married or in a union. Regarding employment, over half (62.4%) were engaged in housekeeping or home-based work. The average household consisted of about five members, and a notable portion (58.5%) of the households was classified as part of the highest socioeconomic status quintile.

A large majority, 84.0%, of mothers indicated that they had received nutritional advice during their pregnancies, and 80.1% attended four or more antenatal care sessions. The delivery method for most births was vaginal (84.2%), with a mean birth weight of around 3200 g. Notably, about 9.4% of the children had a history of low birth weight. In terms of health, 36.0% of the children experienced morbidity in the past two weeks, with cough being the most reported symptom at 27.0%. Diarrhea and fever were also reported, affecting 11% of the children.

### 3.2. Dietary Diversity, Depression, Male Partner Support, and Breastfeeding Self-Efficacy

Table 2 provides insights into the dietary habits of the study population. A vast majority of participants (97.6%) reported consuming staple foods such as grains and tubers, while dairy products were consumed by 62.9%. However, the intake of pulses was significantly lower at 24.9%. The consumption of meat, poultry, and fish was high, with 90.2% reporting regular intake. Egg consumption was reported to be 21.5%, and the intake of dark green leafy vegetables and various fruits varied widely among participants, indicating a range of dietary diversity. The mean dietary diversity score was 5.44, with 22.4% of participants exhibiting inadequate diversity in their diets.

In terms of mental health, the prevalence of possible depression (EPDS ≥ 10) was 12.4%, with a smaller percentage (5.7%) showing probable major depression (EPDS ≥ 13). The average scores for Breastfeeding Self-Efficacy and male partner support were 48.04 and 23.04, respectively.

The results show that 45 out of 793 participants reported probable major depression (Table 2). Of those who did not plan or want their birth, 7.43% experienced depression. Meanwhile, only 4.29% of those who planned or wanted their birth showed signs of depression. This suggests that birth planning may reduce the risk of major depressive symptoms postpartum (*p* = 0.058), even if the *p*-value was slightly over the alpha set for this study (results not presented in tables).

### 3.3. Exclusive Breastfeeding Prevalence

Figure S1 presents the estimated percentage of infants who were exclusively breastfed according to the survey for each of the three measures described above. The proportion of infants in the study sample who were exclusively breastfed was 29.1% (95% CI: 26.0–32.3%) using EBF-24H, 34.9% (95% CI: 31.4–38.6%) according to the “self-declaration”, and 25.1% (95% CI: 22.2–28.2%) using EBF-SB. Exclusive breastfeeding was validated across all three metrics for 190 (24%) infants, underscoring a robust concordance across the variables. Ultimately, 509 records (64%) did not indicate exclusive breastfeeding through any of the three metrics.

### 3.4. Factors Associated with Exclusive Breastfeeding and Breastfeeding Self-Efficacy Score in Bivariate Analysis

Table 3 reveals no significant association between the maternal age, marital status, occupation, household size, and socioeconomic level with exclusive breastfeeding. Nonetheless, the educational level exhibited a statistically significant link (*p* = 0.023), indicating higher exclusive breastfeeding rates among individuals with lower educational attainment. Religious affiliation exhibited significance (*p* = 0.002), indicating that participants from the Evangelical Church reported elevated rates of exclusive breastfeeding in comparison to other religious groups. Child factors, especially age, were significantly impactful (*p* < 0.001), with younger newborns (0–1 month) demonstrating higher exclusive breastfeeding rates than older infants. Morbidity in the preceding two weeks markedly decreased exclusive breastfeeding rates, particularly in cases of cough and fever, which correlated with diminished EBF rates among the impacted children (*p* < 0.001 and *p* = 0.002, respectively). The results indicate that sociodemographic factors, in conjunction with health circumstances, significantly influence exclusive breastfeeding behaviors.

Mothers of exclusively breastfed infants possess a greater dietary diversity score, averaging 5.62, compared to 5.40 for those whose infants are not exclusively breastfed; nonetheless, the difference is only marginally significant (*p* = 0.056). The mother’s self-efficacy score is markedly elevated for exclusively breastfed newborns, averaging 49.62, compared to 47.63 for non-exclusively breastfed infants (*p* < 0.001). The husband’s support in exclusive breastfeeding score is significantly higher for infants who are exclusively breastfed, averaging 24.32, compared to 22.63 for those who are not exclusively nursed (*p* = 0.002). The data also suggests that mothers in the higher quintile experience significantly greater confidence in their breastfeeding abilities than those in the middle quintile (Figures S2–S4).

### 3.5. Factors Associated with Exclusive Breastfeeding and Breastfeeding Self-Efficacy Using SEM

**Figure 1:**
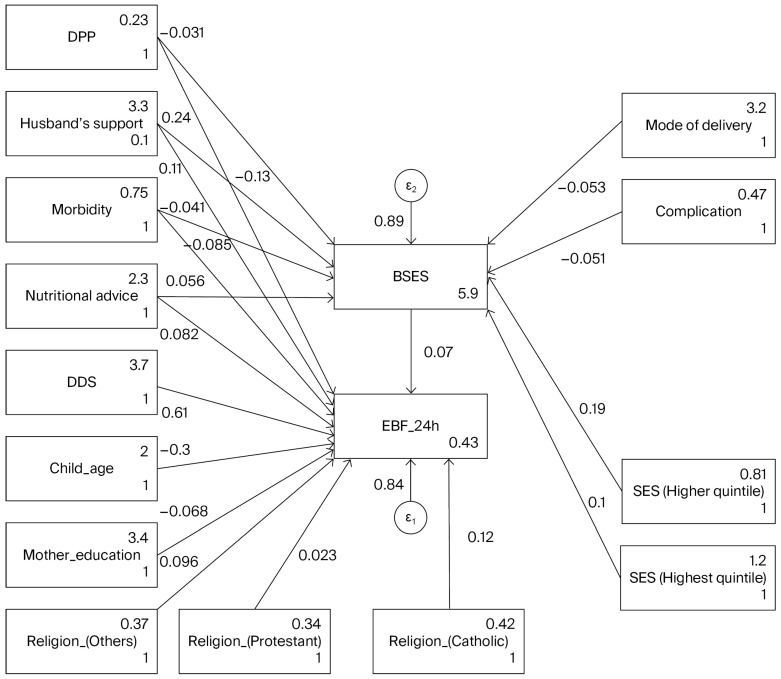
SEM model among entire sample showing standardized coefficient.

This analysis utilized structural equation modeling (see Table 4 and Figure 1) to investigate the associated factors of exclusive breastfeeding at 24 h and breastfeeding self-efficacy ratings across 793 mother–child pairs. The model was calibrated using maximum likelihood estimation, with missing data addressed via the maximum likelihood with missing values approach. The Breastfeeding Self-Efficacy score is positively influenced by nutritional advice during pregnancy, with a coefficient of 2.17, indicating that improved education in nutrition significantly correlates with increased breastfeeding support (*p* = 0.0028). However, child morbidity in the last 2 weeks showed a negative association with the Breastfeeding Self-Efficacy score (coefficient = −1.93, *p* < 0.001), implying that higher morbidity rates are associated with lower breastfeeding support. The husband’s support in exclusive breastfeeding positively correlates with the Breastfeeding Self-Efficacy score (coefficient = 0.23, *p* < 0.001). A significant negative relationship exists between child age and EBF (coefficient = −0.095, *p* < 0.001), suggesting that as the child ages, exclusive breastfeeding rates may decline. Conversely, religion positively influences EBF, indicating that cultural and religious contexts play an important role in breastfeeding practices. The EBF is positively influenced by nutritional advice during pregnancy, with a coefficient of 0.12, indicating that improved education in nutrition significantly correlates with increased EBF practice (*p* = 0.016). Child morbidity in the last 2 weeks showed a negative association with EBF practice (coefficient = −0.09, *p* = 0.014). There is a noteworthy negative association of the mother’s education with EBF (coefficient = −0.118, *p* = 0.068); however, the *p*-value did not reach the significance threshold of 5%. The association between EBF or BSES and PPD as a theoretically relevant variable was not supported by our data.

Post-estimation diagnostics revealed an adequate model fit, demonstrated by an R-squared value of 0.24 for the overall model, with goodness-of-fit statistics indicating potential areas for improvement. Exploring further variables or reassessing the model framework may enhance predictive accuracy. Model statistics showed that the model adequately fit the data (RMSEA = 0.11, CFI = 0.987, TLI = 0.963). The Akaike Information Criterion (AIC) and Bayesian Information Criterion (BIC) indicate that although the model demonstrates a satisfactory fit, further refinement is required.

In a sensitivity analysis, when comparing complete-case and MICE approaches, the MICE methodology frequently uncovers subtle correlations that may be concealed in whole-case analyses, offering a more thorough comprehension of the determinants affecting exclusive breastfeeding. Consequently, employing MICE not only strengthens the validity of the findings but also augments the clarity of the data, finally facilitating improved actions for advancing EBF. The negative association between maternal education and exclusive breastfeeding was not robust after multiple imputation (Table S2).

## 4. Discussion

In this study, we estimated the prevalence of exclusive breastfeeding in three ways among the same populations and identified key factors associated with breastfeeding. The EBF status based on a one-question self-declaration by the mother resulted in the highest estimates. The EBF-24H indicator was higher than the EBF-SB indicator. Several factors had an association: (1) failure to capture prelacteal feeding; (2) failure to capture intermittent use of complementary feeds; and (3) the assumption that the feeding pattern at the time of the survey will continue until 6 months of age [40,41,42]. EBF-SB is more sensitive to the first two issues; that is, it can capture both prelacteal feeding and the intermittent use of complementary feeds. Using EBF-SB produces results lower than those of the other two methods. The prevalence of EBF reported in this study is much lower than the national estimate reported by the most recent DHS in the DRC [5]. Urban Kinshasa presents distinctive breastfeeding challenges that may be exacerbated by various socioeconomic factors. The rapid urbanization of the city has led to increased employment constraints, where many mothers work long hours and may lack flexible work arrangements that accommodate breastfeeding. Additionally, the commercialization and aggressive marketing of formula milk and complementary foods in urban settings undermine traditional breastfeeding practices, making it easier for mothers to rely on substitutes [13,14]. Urban stressors, such as overcrowding, high living costs, and limited access to healthcare resources, further complicate the breastfeeding landscape. Migration patterns also play a role, as many families move to urban areas seeking better opportunities, often leaving behind established support networks that are crucial for successful breastfeeding. These multifaceted challenges necessitate targeted interventions that consider the unique context of urban Kinshasa to promote and sustain breastfeeding practices effectively.

Given these challenges, it is crucial to explore how enhancing mothers’ confidence in their breastfeeding abilities can mitigate some of these issues. Breastfeeding self-efficacy refers to a mother’s confidence in her ability to breastfeed successfully. Our results indicate that higher levels of self-efficacy are associated with an increased likelihood of exclusive breastfeeding. This aligns with existing works in the literature suggesting that mothers who believe in their ability to breastfeed are more likely to initiate and maintain breastfeeding practices [25,43]. Enhancing breastfeeding self-efficacy can be achieved through targeted interventions such as prenatal education programs, where mothers receive information and support focused on breastfeeding techniques and overcoming common challenges. Peer support groups and mentorship programs can also play a pivotal role in reinforcing mothers’ confidence and ensuring they feel supported throughout their breastfeeding journey [44]. It should be noted that, as shown by the results of this study, the role of the husband/male partner’s support has emerged as a critical associated factor in breastfeeding self-efficacy. Our study found that when husbands are actively involved and supportive, mothers report higher levels of confidence in their breastfeeding abilities. This finding is consistent with research that highlights the positive impact of paternal involvement on maternal health outcomes [45]. Husbands can provide emotional support and practical assistance, which can alleviate stress and encourage mothers to persist with breastfeeding, especially during challenging times. Therefore, it is essential to include fathers in prenatal education and breastfeeding support programs. Engaging fathers not only strengthens family bonds but also creates an environment conducive to breastfeeding success. When considering prenatal education and breastfeeding support programs, there is a need to think differently about current practices regarding nutritional advice during pregnancy. Nutritional advice during pregnancy was also positively associated with breastfeeding self-efficacy in our study. Proper nutrition is fundamental for mothers, as it is associated with both their health and their capacity to produce milk. Research has shown that women who receive adequate nutritional guidance are more likely to feel empowered and capable of breastfeeding successfully [46]. Healthcare providers should prioritize nutritional counseling as an integral component of prenatal care. Providing mothers with information about dietary choices that support breastfeeding can enhance their confidence and commitment to exclusive breastfeeding. Furthermore, community-based programs that focus on educating mothers about the nutritional aspects of breastfeeding can foster a supportive environment that encourages healthy practices.

Improving breastfeeding self-efficacy will improve EBF practice, as shown in this study. The findings of this study reveal significant relationships between exclusive breastfeeding (EBF) and several key factors, including breastfeeding self-efficacy, the maternal education level, husband support, nutritional advice during pregnancy, the child’s age, morbidity in the last two weeks, and religion. Understanding these associations is crucial for developing comprehensive strategies to promote EBF, ultimately enhancing infant health outcomes. Our results indicate a strong correlation between breastfeeding self-efficacy and the likelihood of maintaining EBF. This underscores the importance of empowering mothers with confidence in their breastfeeding abilities. Interventions that focus on enhancing self-efficacy—such as prenatal education sessions and peer support groups—could play a pivotal role in improving EBF rates. When mothers believe in their capacity to breastfeed successfully, they are more likely to persevere through challenges, leading to longer durations of EBF [25,43,47]. The study also highlights the association of maternal education and EBF practices, showing a noteworthy negative association, although the *p*-value did not reach the significance threshold of 5%. The observed association between maternal education and exclusive breastfeeding should be interpreted cautiously as it was not robust after multiple imputations. This association was unstable and could be context dependent. This could suggest that educational levels may inversely affect exclusive breastfeeding practices, possibly due to varying perceptions, professional occupation, and priorities regarding breastfeeding among educated mothers. Enhanced female education correlates with heightened labor force involvement [48]. Resuming employment is correlated with suboptimal breastfeeding behaviors [49,50]. As women’s formal education and labor force participation rise, protective regulations are consequently essential to supporting breastfeeding. However, such enabling policies are absent in most nations, and even when enacted, their implementation is insufficient, leading to limited coverage. While the previous literature suggested that lower educational levels can negatively affect breastfeeding practices [51], our study’s findings may not reflect this trend due to the under-representation of this group. Indeed, the small sample size of mothers with low educational attainment may limit our ability to generalize findings regarding their influence on breastfeeding practices. In the present study, mothers with a low educational level (6.9%) were under-represented compared to the majority in higher educational categories. Educated mothers are generally more aware of the benefits of breastfeeding and are better equipped to navigate potential barriers. This finding emphasizes the need for targeted educational programs that inform and equip mothers with the knowledge necessary to sustain EBF. Policies aimed at increasing educational opportunities for women could have a cascading association with improving breastfeeding rates within communities [3,52].

Support from husbands and nutritional guidance during pregnancy directly affected exclusive breastfeeding (EBF) in this study, either independently or through Breastfeeding Self-Efficacy Scale (BSES) influences. The role of husband support was identified as a significant factor in EBF, its effect on exclusive breastfeeding appears mainly indirect, operating through self-efficacy, highlighting the importance of husbands/male partner involvement in breastfeeding practices. Partner support can help reduce stress and provide essential emotional encouragement during the challenging postpartum period. Initiatives that involve fathers in both prenatal and postnatal education can create a supportive atmosphere conducive to promoting breastfeeding. Encouraging active paternal involvement not only enhances maternal well-being but also strengthens family dynamics [45,46]. The provision of nutritional advice during pregnancy was positively associated with EBF. Access to proper nutrition and guidance can empower mothers to make informed dietary choices that support their health and breastfeeding efforts. Healthcare providers should prioritize nutritional counseling as part of routine prenatal care, ensuring that mothers receive the necessary information to optimize their health and breastfeeding success [53,54].

It was also found in this study that child age significantly influenced EBF practices, as younger infants are more likely to be exclusively breastfed. Additionally, higher morbidity in the last two weeks was associated with a decreased likelihood of EBF, suggesting that health complications can hinder breastfeeding efforts. This relationship highlights the importance of monitoring infant health and providing appropriate medical care to support continued breastfeeding. Pediatric healthcare providers should advocate for and support breastfeeding, particularly during periods of illness [55,56]. Finally, the role of religion in shaping breastfeeding practices warrants further exploration. Cultural and religious beliefs can significantly influence maternal attitudes towards breastfeeding. Understanding the religious contexts and values surrounding breastfeeding can inform culturally sensitive interventions that respect these beliefs and integrate them into educational programs [57,58].

### 4.1. Strengths and Limitations

This study has several limitations. It utilizes a cross-sectional methodology, which restricts the capacity to determine causal links among independent variables, and exclusive breastfeeding practices. Causal inference is not possible due to the cross-sectional design. This study did not demonstrate a correlation between depression and the two outcomes (EBF and breastfeeding self-efficacy); an extensive study with a larger sample size is necessary to determine whether this relationship exists in Kinshasa, particularly considering the high incidence of unwanted pregnancies [21,59], which also maybe associated with postpartum depression, as previously noted. PPD as a theoretically relevant variable was not supported by our data. Longitudinal studies are essential for comprehending the temporal dynamics and causality of these connections. Dependence on self-reported questionnaires may introduce bias, as mothers may underreport symptoms of postpartum depression or exaggerate their breastfeeding practices according to social desirability. Future research may benefit from the incorporation of clinical assessments in mental health evaluations. The sample size, although sufficient for the initial findings, may not comprehensively reflect the heterogeneous population of mothers in Kinshasa or other areas of the DRC. Outcomes may also change through various socioeconomic or cultural circumstances. The evaluation of dietary diversity failed to consider specific nutrient intake or the quality of the foods ingested. A comprehensive investigation of food habits could yield further insights into the correlation between maternal nutrition and nursing. The assessment of dietary diversity is based on a single 24 h recall, which primarily captures short-term dietary intake rather than habitual consumption patterns. We caution readers to interpret the findings with this limitation in mind, recognizing that the results may not fully reflect long-term dietary behaviors. Although we endeavored to account for numerous confounding variables, there may be unmeasured factors (such as social support and health literacy) that could affect both maternal mental health and breastfeeding results. The cultural attitudes and behaviors related to breastfeeding and maternal mental health were not thoroughly examined, outside of religion. These factors can have a profound impact on breastfeeding patterns and may limit the generalizability of the findings across various cultural contexts. The study occurred within a defined chronological framework, where exogenous variables such as political instability or health emergencies (e.g., the mpox epidemic, flooding, and socioeconomic crises) could have impacted maternal mental health and breastfeeding behaviors during this timeframe. Another significant limitation of this study is the use of a non-standardized tool to measure the husband’s support for exclusive breastfeeding. The husband support tool has not undergone thorough validation, and thus, the construct validity remains uncertain. While the tool was developed to capture relevant dimensions of support, its lack of validation and standardization may affect the reliability and comparability of the results, such as the paternal support scale of breastfeeding [60]. Consequently, we have interpreted the findings derived from this tool as exploratory in nature. This limitation raises concerns regarding the accuracy of the assessments and the potential for measurement bias, as the tool may not fully encompass the complexities of spousal support or the various ways in which it can influence breastfeeding practices. Future research should consider employing validated instruments to measure familial support, ensuring more robust and generalizable findings.

We acknowledge the limitation regarding self-reported data for the question “Did you feed your child only breast milk yesterday?” While our study only included mothers as participants to minimize bias from other caregivers, it is possible that some respondents may not have been biological mothers but rather other caregivers. This introduces a potential for misreporting, as these individuals may not have complete knowledge of the child’s feeding practices. Future research should consider employing more robust verification methods to accurately assess feeding behaviors and account for the roles of all caregivers involved.

This study makes several significant contributions to the understanding of breastfeeding practices, with a particular focus on the interplay between breastfeeding self-efficacy, husband support, and nutritional advice during pregnancy. These contributions are vital for both academic discourse and practical applications in maternal and child health. By examining the direct relationship between breastfeeding self-efficacy and its contributing factors, this study enriches the existing framework on breastfeeding practices. It provides empirical evidence that empowers healthcare practitioners to develop targeted interventions aimed at increasing self-efficacy among mothers. This can lead to higher rates of exclusive breastfeeding, which is crucial for infant health. This research underscores the critical role of the husband’s support in breastfeeding journeys. By identifying the husband’s support as a significant predictor of breastfeeding self-efficacy, the study advocates for the inclusion of fathers in prenatal education and breastfeeding support initiatives. This contribution not only broadens the scope of the existing literature but also promotes a more inclusive approach to maternal health, recognizing the family unit’s role in successful breastfeeding practices. The findings emphasize the importance of nutritional advice during pregnancy as an associated factor for breastfeeding self-efficacy. This contribution highlights the need for healthcare providers to integrate or strengthen nutritional counseling into routine prenatal care. By focusing on the nutritional needs of expectant mothers, the study advocates comprehensive strategies that support mothers in their breastfeeding efforts, ultimately benefiting both maternal and infant health. In this study, we utilize the mother–child dyad as the unit of analysis, a relatively novel approach in breastfeeding research. This methodology allows for a more holistic and multidimensional understanding of breastfeeding practices by capturing the intricate dynamics between maternal factors and child outcomes. By focusing on the interactions within the dyad, we can better explore how maternal psychological well-being, social support, and sociodemographic factors collectively influence exclusive breastfeeding behaviors, ultimately enhancing our insights into effective interventions and support mechanisms. In recognizing breastfeeding as a collective accomplishment, this study emphasizes that EBF outcomes are influenced not only by maternal and infant factors but also by the social and environmental contexts surrounding them. By framing our variables within this collaborative perspective, we highlight the importance of support systems, including family, healthcare providers, and community resources, that contribute to successful EBF practices. This approach underscores the need for comprehensive strategies that engage all stakeholders in promoting positive breastfeeding outcomes. The MICE approach enhances statistical analysis by addressing biases from missing data, unlike complete-case analysis, which may misrepresent factors influencing exclusive breastfeeding. It includes all observations, thereby improving statistical power and revealing significant associations, such as the pronounced effect of the “husband’s support in exclusive breastfeeding score.” MICE captures complex relationships, showcasing the importance of “nutritional advice during pregnancy” and demonstrating critical findings like the negative impact of “morbidity in the last 2 weeks.” The methodology provides consistent effect estimates for predictors, contrasting with the variability seen in complete-case analysis that can lead to misinterpretations.

### 4.2. Practical Implications for Healthcare Providers

The study’s insights offer practical implications for healthcare providers, policymakers, and maternal health programs. By identifying key factors that enhance breastfeeding self-efficacy, the research can inform the design of education and support programs tailored to meet the needs of mothers. This aligns with current public health goals to increase breastfeeding rates and improve maternal and child health outcomes.

### 4.3. Suggestions for Future Research

This study lays the groundwork for future research exploring the dynamics of breastfeeding support systems. It opens avenues for further investigation into how various familial and social factors influence breastfeeding practices, paving the way for more nuanced and effective intervention strategies. In light of the insights gained from this study, we recommend that future research should include follow-up data collection to better identify causal relationships between the identified variables and exclusive breastfeeding outcomes. Longitudinal studies could provide a more robust understanding of how these factors interact over time. Furthermore, employing multi-level modeling techniques could enhance the explanatory power of future investigations by allowing researchers to account for the hierarchical nature of the data, including individual, familial, and community-level influences on breastfeeding practices. This approach would facilitate a more nuanced analysis of the complex interplay of factors affecting exclusive breastfeeding.

## 5. Conclusions

This study highlights the multifaceted challenges faced by mothers in Kinshasa regarding exclusive breastfeeding. The findings of the current study can assist health professionals and decision-makers in designing and implementing culturally tailored interventions to enhance maternal breastfeeding self-efficacy. The findings of this study reveal significant relationships between breastfeeding self-efficacy, husband support, and nutritional advice during pregnancy. These factors are crucial in promoting EBF and can significantly influence maternal and infant health outcomes. By prioritizing the roles of husband support and nutritional advice during pregnancy, we can create a more supportive framework for breastfeeding practices. Future research should explore longitudinal approaches to elucidate the roles of husband support and nutritional advice during pregnancy on breastfeeding and infant health, as well as the potential benefits of integrated maternal health programs that address nutritional needs.

## Figures and Tables

**Table 1:** Characteristics of mothers and children.

Characteristics	n	%
Mother’s characteristics		
Age (in years) (mean ± SD)	28.74 ± 5.72 years	
18–24	191	24.1
25–34	466	58.8
35–49	136	17.2
Education level		
Low	55	6.9
Secondary school or above	738	93.1
Marital status		
Single	101	12.7
Married/in union	692	87.3
Occupation		
Occupation outside the household	298	37.6
Housekeeping or other home occupation	495	62.4
Religion		
Evangelical Church	513	64.7
Catholic	109	13.7
Protestant	81	10.2
Other	90	11.3
Household size (mean ± SD)	5.34 ± 2.23	
≤6	594	74.9
>6	199	25.1
Parity		
Primiparous	356	44.9
Pauciparous	311	39.2
Multiparous	126	15.9
Intergenetic space		
<2 years	186	42.6
≥2 years	251	57.4
Number of antenatal care sessions attended		
None	5	0.6
1 to 3	153	19.3
4 or more	635	80.1
Mode of delivery		
Vaginal delivery	668	84.2
Cesarean section	125	15.8
Complication during pregnancy or childbirth	151	19.0
Nutritional advice during pregnancy	666	84.0
Planned or wanted birth	443	55.9
Child characteristics	n	%
Age (in months) (mean ± SD)	2.12 ± 0.69	
0–1 month	140	18.4
2–3 months	387	50.8
4–5 months	235	30.8
Sex		
Girl	385	48.5
Boy	408	51.5
Birth weight (mean ± SD)	(3197.89 ± 572.45 g)	
BW < 2500 g	75	9.4
BW ≥ 2500 g	721	90.6
Morbidity in the last 2 weeks	224	36.0
Diarrhea	68	10.9
Cough	168	27.0
Fever	69	11.1
Household characteristics		
Socioeconomic status		
Middle quintile	26	3.3
Higher quintile	303	38.2
Highest quintile	464	58.5

SD: standard deviation, BW: birth weight.

**Table 2:** Distribution of food group consumption, depression, male partner support, and Breastfeeding Self-Efficacy score.

	n	%
Grains, white roots and tubers, and plantains	777	97.6
Pulses (beans, peas, and lentils)	198	24.9
Nuts and seeds	363	45.6
Dairy	501	62.9
Meat, poultry, and fish	718	90.2
Eggs	171	21.5
Dark green leafy vegetables	536	67.3
Other vitamin A-rich fruits and vegetables	690	86.7
Other vegetables	84	10.6
Other fruits	294	36.9
Dietary diversity score (mean ± SD)	5.44 ± 1.48	
MDD_W		
Inadequate diversity	178	22.4
Adequate diversity	618	77.6
Edinburgh Postnatal Depression		
EPDS * (median (IQR))	3 (Q1 = 1, Q3 = 7)	
EPDS * ≥ 10 (possible depression)	99	12.4
EPDS * ≥ 13 (probable major depression)	45	5.7
Breastfeeding Self-Efficacy score (mean ± SD)	48.04 ± 7.55	
Tertile of Breastfeeding Self-Efficacy score		
Low	287	36.1
Medium	296	37.2
High	213	26.8
Husband’s support in exclusive breastfeeding score (mean ± SD)	23.04 ± 7.11	
Tertile of husband’s support in exclusive breastfeeding score		
Low	293	36.8
Medium	254	31.9
High	249	31.3
Received “nutrition advice during pregnancy”	317	40.0

* Edinburgh Postnatal Depression Scale (EPDS). SD: standard deviation. Q1: first quartile, Q3: third quartile. IQR: interquartile range.

**Table 3:** Association between exclusive breastfeeding and participants’ sociodemographic and economic variables in bivariate analysis.

	EBF Using EBF-24H				*p*-Value
	No		Yes		
	n	%	n	%	
Mother’s age (in years)					0.530
18–24	140	25.0	50	21.7	
25–34	323	57.6	142	61.7	
35–49	98	17.5	38	16.5	
Education level					0.023
Low	31	5.5	23	10.0	
Secondary school or above	530	94.5	207	90.0	
Marital status					0.882
Single	71	12.7	30	13.0	
Married/in union	490	87.3	200	87.0	
Occupation					0.955
Occupation outside the household	211	37.6	87	37.8	
Housekeeping or other home occupation	350	62.4	143	62.2	
Religion					0.002
Evangelical Church	384	68.4	128	55.7	
Catholic	67	11.9	42	18.3	
Protestant	57	10.2	24	10.4	
Other	53	9.4	36	15.7	
Household size					0.156
≤6	412	73.4	180	78.3	
>6	149	26.6	50	21.7	
Socioeconomic status					0.782
Middle quintile	19	3.4	7	3.0	
Higher quintile	218	38.9	84	36.5	
Highest quintile	324	57.8	139	60.4	
Parity					0.098
Primiparous	257	45.8	97	42.2	
Pauciparous	208	37.1	103	44.8	
Multiparous	96	17.1	30	13.0	
Intergenetic space					0.583
<2 years	132	43.4	54	40.6	
≥2 years	172	56.6	79	59.4	
Number of prenatal consultations attended					0.858
None	3	0.5	2	0.9	
1 to 3	108	19.3	45	19.6	
4 or more	450	80.2	183	79.6	
Mode of delivery					0.869
Vaginal delivery	473	84.3	195	84.8	
Cesarean section	88	15.7	35	15.2	
Nutritional education	459	81.8	205	89.1	0.011
Planned or wanted birth	309	55.1	132	57.4	0.552
Child characteristics					
Child age (in months)					<0.001
0–1	76	14.3	64	27.8	
02–03	256	48.3	130	56.5	
04–05	198	37.4	36	15.7	
Sex					0.352
Girl	279	49.7	106	46.1	
Boy	282	50.3	124	53.9	
Birth weight					0.069
BW < 2500 g	60	10.7	15	6.5	
BW ≥ 2500 g	501	89.3	215	93.5	
Morbidity in the last 2 weeks *	169	41.5	55	25.7	<0.001
Diarrhea	50	12.3	18	8.4	0.142
Cough	127	31.2	41	19.2	<0.001
Fever	57	14.0	12	5.6	0.002
EPDS ≥ 10 (possible depression)	78	13.9	21	9.1	0.065
EPDS ≥ 13 (probable major depression)	38	6.8	7	3.0	0.040
Dietary diversity score (mean ± SD)	5.40 ± 1.44		5.62 ± 1.47		0.0562
Breastfeeding Self-Efficacy score (mean ± SD)	47.63 ± 0.30		49.62 ± 0.37		0.0002
Husband’s support in exclusive breastfeeding score (mean ± SD)	22.63 ± 6.78		24.32 ± 7.33		0.0020

* Reported at least one of the three symptoms: diarrhea, fever, or cough. SD: standard deviation. BW: birth weight.

**Table 4:** Direct and indirect associations of various factors with exclusive breastfeeding.

Variables	Direct Effect			Indirect Effect			Total Effect		
	Coef. **	Std. Err.	*p*	Coef. **	Std. Err.	*p*	Coef. **	Std. Err.	*p*
EBF using EBF-24H									
Breastfeeding Self-Efficacy score	0.005	0.003	0.0949	0.000	0.000		0.005	0.003	0.0949
Child age (in months)	−0.095	0.012	0.0000	0.000	0.000		−0.095	0.012	0.0000
Religion (Catholic vs. Evangelical Church)	0.154	0.051	0.0025	0.000	0.000		0.154	0.051	0.0025
Religion (Protestant vs. Evangelical Church)	0.037	0.060	0.5353				0.037	0.060	0.5353
Religion (Other vs. Evangelical Church)	0.140	0.056	0.0124				0.140	0.056	0.0124
Dietary diversity score	0.020	0.012	0.0949	0.000	0.000		0.020	0.012	0.0949
Nutritional advice during pregnancy	0.107	0.049	0.0293	0.010	0.007	0.1527	0.118	0.049	0.0160
Morbidity in the last 2 weeks *	−0.084	0.038	0.0271	−0.009	0.006	0.1336	−0.093	0.038	0.0143
Husband’s support in exclusive breastfeeding score	0.004	0.003	0.1835	0.001	0.001	0.3173	0.005	0.003	0.0949
EPDS ≥ 13 (probable major depression)	−0.087	0.081	0.2846	−0.005	0.006	0.4065	−0.092	0.082	0.2627
Mother’s education level	−0.118	0.065	0.0688	0.000	0.000		−0.118	0.065	0.0688
Mode of delivery	0.000	0.000		−0.005	0.004	0.2113	−0.005	0.004	0.2113
Complication during pregnancy or childbirth	0.000	0.000		−0.004	0.004	0.3173	−0.004	0.004	0.3173
Socioeconomic status (higher vs. middle)	0.000	0.000		0.013	0.010	0.1936	0.013	0.010	0.1936
Socioeconomic status (highest vs. middle)	0.000	0.000		0.007	0.008	0.3789	0.007	0.008	0.3789
Breastfeeding Self-Efficacy Score									
Nutritional advice during pregnancy	2.170	0.725	0.0028	0.000	0.000		2.170	0.725	0.0028
Morbidity in the last 2 weeks *	−1.927	0.548	0.0004	0.000	0.000		−1.927	0.548	0.0004
Husband’s support in exclusive breastfeeding score	0.231	0.038	0.0000	0.000	0.000		0.231	0.038	0.0000
EPDS >= 13 (probable major depression)	−0.963	1.215	0.4295	0.000	0.000		−0.963	1.215	0.4295
Mode of delivery	−0.998	0.737	0.1770	0.000	0.000		−0.998	0.737	0.1770
Complication during pregnancy or childbirth	−0.905	0.706	0.2005	0.000	0.000		−0.905	0.706	0.2005
Socioeconomic status (higher vs. middle)	2.752	1.505	0.0672				2.752	1.505	0.0672
Socioeconomic status (highest vs. middle)	1.460	1.496	0.3271	0.000	0.000	.	1.460	1.496	0.3271

* Reported at least one of the three symptoms: diarrhea, fever, or cough. ** Unstandardized coefficient.

## Data Availability

A de-identified dataset and codebook are available at OSF (https://doi.org/10.17605/OSF.IO/CSJF4, accessed on 15 December 2025. Any additional materials that cannot be shared openly due to ethical restrictions are available from KSPH upon reasonable request.

## Supplementary Materials

The following supporting information can be downloaded at https://www.mdpi.com/article/10.3390/nu18010065/s1: Figure S1: Prevalence of exclusive breastfeeding by metric; Figure S2: Relationship between breastfeeding self-efficacy score and dietary diversity score (a) and husband’s support in exclusive breastfeeding score (b); Figure S3: Breastfeeding self-efficacy by mother’s characteristics; Figure S4: Breastfeeding self-efficacy by mother’s characteristics; Figure S5: Breastfeeding self-efficacy by mother’s characteristics; Table S1: Male partner/husbands’ support for exclusive breastfeeding; Table S2: Comparative analysis of direct and indirect associations of various factors on exclusive breastfeeding: complete-case versus multiple imputation approaches.
