# Supplementary material for: Understanding the Interplay of Maternal Mental Health, Social Support, and Sociodemographic Factors in Promoting Exclusive Breastfeeding in Kinshasa
Source: Nutrients. 2025 Dec 25;18(1):65. doi: 10.3390/nu18010065 (PMC12787543; doi:10.3390/nu18010065)
Supplement: Supplementary file 1 [file nutrients-18-00065-s001.zip › english-104458-tables-S1,S2.pdf]

nutrients

MDPI

Table S1. Male partner/husbands' support for exclusive breastfeeding.

1. On a scale of 1 to 5, how would you rate your husband's emotional support during breastfeeding?	1. Not at all supportive<br>2. A little<br>3. Moderately<br>4. Strongly<br>5. Very supportive
2. How often does your husband encourage you to breastfeed	1. Never<br>2. Rarely<br>3. Sometimes<br>4. Often<br>5. Always
3. How often does your husband take care of the other children while you are breastfeeding?	1. Never<br>2. Rarely<br>3. Sometimes<br>4. Often<br>5. Always
4. On a scale of 1 to 5, how would you rate your husband's knowledge of breastfeeding?	1. Very poor<br>2. Poor<br>3. Neutral<br>4. Good<br>5. Very good
5. On a scale of 1 to 5, how much do you think your husband values breastfeeding?	1. Not at all<br>2. A little<br>3. Moderately<br>4. Strongly<br>5. Very strongly
6. How often do you discuss breastfeeding challenges with your husband?	1. Never<br>2. Rarely<br>3. Sometimes<br>4. Often<br>5. Always
7. On a scale of 1 to 5, how much does your husband's support influence your decision to breastfeed?	1. Not at all<br>2. A little<br>3. Moderately<br>4. Strongly<br>5. Very strongly

Table S2. Comparative analysis of direct & indirect effects of various factors on exclusive breastfeeding: complete case versus multiple imputation approaches.

Variables	Complete Cases									Multiple Imputation Approaches								
	Direct Effect			Indirect Effect			Total Effect			Direct Effect			Indirect Effect			Total Effect		
	Coef**.	Std. Err.	*p*	Coef**.	Std. Err.	*p*	Coef**.	Std. Err.	*p*	Coef**.	Std. Err.	*p*	Coef**.	Std. Err.	*p*	Coef**.	Std. Err.	*p*
**EBF using EBF-24H**																		
Breastfeeding Self-Efficacy Score	0.005	0.003	0.0949	0.000	0.000	.	0.005	0.003	0.0949	0.005	0.003	0.0949	0.000	0.000	.	0.005	0.003	0.0949
Child age (in months)	−0.095	0.012	0.0000	0.000	0.000	.	−0.095	0.012	0.0000	−0.049	0.011	0.0000	0.000	0.000	.	−0.049	0.011	0.0000
Religion (Catholic vs. Evangelic Church)	0.154	0.051	0.0025	0.000	0.000	.	0.154	0.051	0.0025	0.029	0.053	0.5823	0.000	0.000	.	0.029	0.053	0.5823
Religion (Protestant vs. Evangelic Church)	0.037	0.060	0.5353				0.037	0.060	0.5353	0.020	0.050	0.6892	0.000	0.000	.	0.020	0.050	0.6892
Religion (Other vs. Evangelic Church)	0.140	0.056	0.0124				0.140	0.056	0.0124	0.077	0.033	0.0198	0.000	0.000	.	0.077	0.033	0.0198
Dietary diversity score	0.020	0.012	0.0949	0.000	0.000	.	0.020	0.012	0.0949	−0.000	0.009	1.0000	0.000	0.000	.	−0.000	0.009	1.0000
Nutritional advice during pregnancy	0.107	0.049	0.0293	0.010	0.007	0.1527	0.118	0.049	0.0160	0.053	0.043	0.2187	0.013	0.012	0.2801	0.066	0.048	0.1676
Morbidity in the last 2 weeks *	−0.084	0.038	0.0271	−0.009	0.006	0.1336	−0.093	0.038	0.0143	−0.030	0.011	0.0063	−0.010	0.006	0.0949	−0.039	0.013	0.0027
Husband's support in exclusive breastfeeding Score	0.004	0.003	0.1835	0.001	0.001	0.3173	0.005	0.003	0.0949	0.004	0.002	0.0455	0.002	0.001	0.0455	0.006	0.002	0.0027
EPDS >=13 (probable major depression)	−0.087	0.081	0.2846	−0.005	0.006	0.4065	−0.092	0.082	0.2627	−0.033	0.081	0.6818	−0.004	0.012	0.7414	−0.036	0.086	0.6745
Mother education level	−0.118	0.065	0.0688	0.000	0.000	.	−0.118	0.065	0.0688	−0.116	0.073	0.1118	0.000	0.000	.	−0.116	0.073	0.1118
Mode of delivery	0.000	0.000	.	−0.005	0.004	0.2113	−0.005	0.004	0.2113	0.000	0.000	.	−0.012	0.014	0.3898	−0.012	0.014	0.3898
Complication during pregnancy/or childbirth	0.000	0.000	.	−0.004	0.004	0.3173	−0.004	0.004	0.3173	0.000	0.000	.	−0.011	0.007	0.1164	−0.011	0.007	0.1164
Socioeconomic status(Higher vs. Middle)	0.000	0.000	.	0.013	0.010	0.1936	0.013	0.010	0.1936	0.000	0.000	.	0.009	0.016	0.5755	0.009	0.016	0.5755
Socioeconomic status(Highest vs. Middle)	0.000	0.000		0.007	0.008	0.3789	0.007	0.008	0.3789	0.000	0.000	.	0.004	0.012	0.7414	0.004	0.012	0.7414
**Breastfeeding Self–Efficacy Score**																		

Nutritional advice during pregnancy	2.170	0.725	0.0028	0.000	0.000	.	2.170	0.725	0.0028	2.350	1.600	0.1416	0.000	0.000	.	2.350	1.600	0.1416
Morbidity in the last 2 weeks *	−1.927	0.548	0.0004	0.000	0.000	.	−1.927	0.548	0.0004	−1.747	0.306	0.0000	0.000	0.000	.	−1.747	0.306	0.0000
Husband's support in exclusive breastfeeding Score	0.231	0.038	0.0000	0.000	0.000	.	0.231	0.038	0.0000	0.340	0.094	0.0003	0.000	0.000	.	0.340	0.094	0.0003
EPDS >= 13 (probable major depression)	−0.963	1.215	0.4295	0.000	0.000	.	−0.963	1.215	0.4295	−0.688	2.237	0.7566	0.000	0.000	.	−0.688	2.237	0.7566
Mode of delivery	−0.998	0.737	0.1770	0.000	0.000	.	−0.998	0.737	0.1770	−2.222	1.267	0.0801	0.000	0.000	.	−2.222	1.267	0.0801
Complication during pregnancy/or childbirth	−0.905	0.706	0.2005	0.000	0.000	.	−0.905	0.706	0.2005	−2.021	0.810	0.0124	0.000	0.000	.	−2.021	0.810	0.0124
Socioeconomic status(Higher vs. Middle)	2.752	1.505	0.0672				2.752	1.505	0.0672	1.716	3.129	0.5823	0.000	0.000	.	1.716	3.129	0.5823
Socioeconomic status(Highest vs. Middle)	1.460	1.496	0.3271	0.000	0.000	.	1.460	1.496	0.3271	0.689	2.306	0.7642	0.000	0.000	.	0.689	2.306	0.7642

*: Reported at least one of the three symptoms: diarrhea, fever, or cough. **: Unstandardized coefficient.
