# Supplementary material for: Understanding the Interplay of Maternal Mental Health, Social Support, and Sociodemographic Factors in Promoting Exclusive Breastfeeding in Kinshasa
Source: Nutrients. 2025 Dec 25;18(1):65. doi: 10.3390/nu18010065 (PMC12787543; doi:10.3390/nu18010065)
Supplement: Supplementary file 1 [file nutrients-18-00065-s001.zip › Figure-S2.pdf]

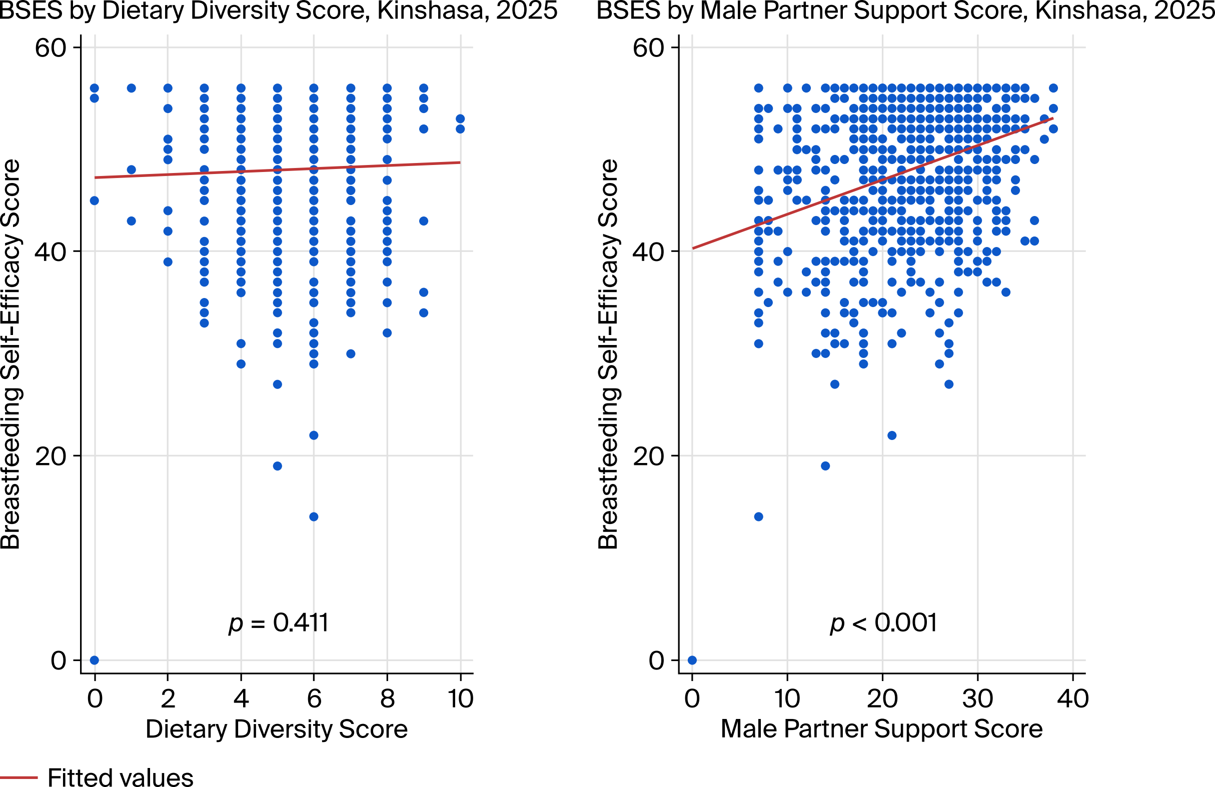
BSES by Dietary Diversity Score, Kinshasa, 2025
BSES by Male Partner Support Score, Kinshasa, 2025
Breastfeeding Self-Efficacy Score
60
40
20
0
p = 0.411
Dietary Diversity Score
0
2
4
6
8
10
p < 0.001
Male Partner Support Score
0
10
20
30
40
Fitted values
