# Supplementary figures and images for: Understanding the Interplay of Maternal Mental Health, Social Support, and Sociodemographic Factors in Promoting Exclusive Breastfeeding in Kinshasa
Source: Nutrients. 2025 Dec 25;18(1):65. doi: 10.3390/nu18010065 (PMC12787543; doi:10.3390/nu18010065)

Venn Diagram  
N = 793

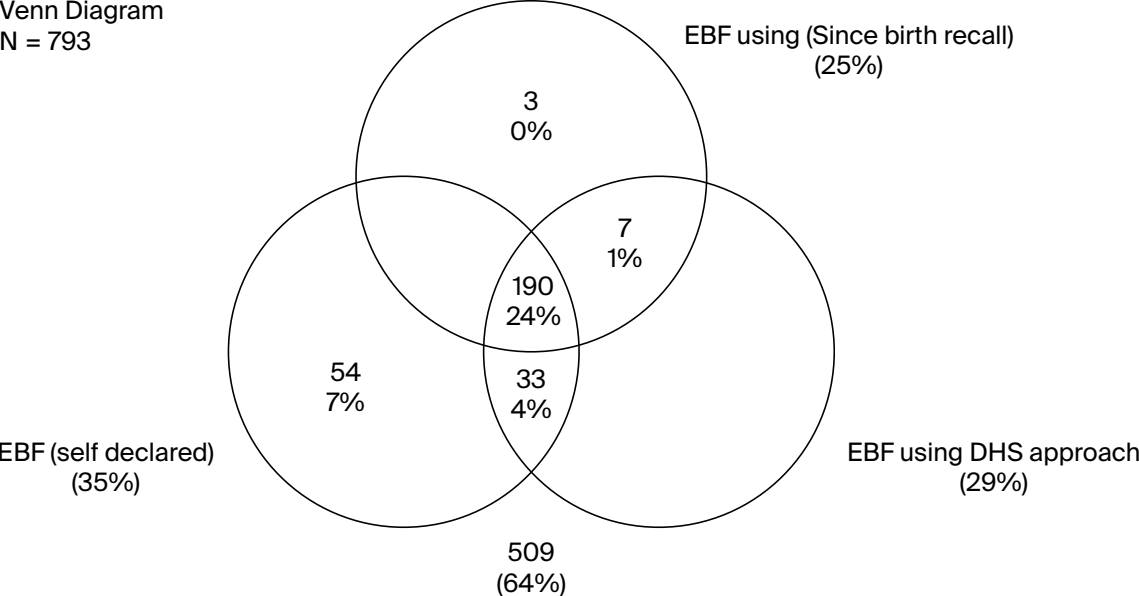

Supplement: Supplementary file 1 [file nutrients-18-00065-s001.zip › Figure-S1.pdf]

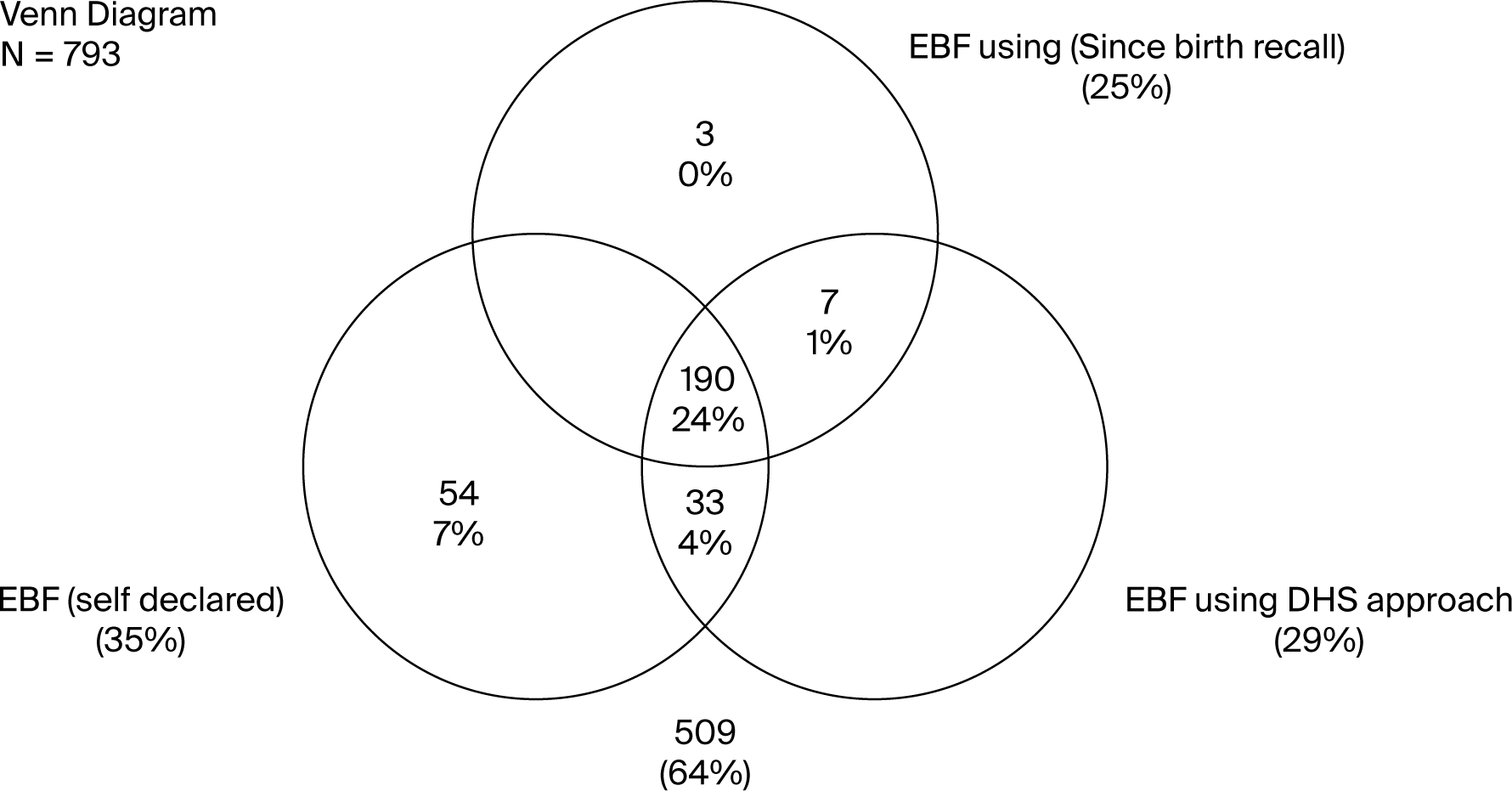

Supplement: Supplementary file 1 [file nutrients-18-00065-s001.zip › Figure-S1.png]

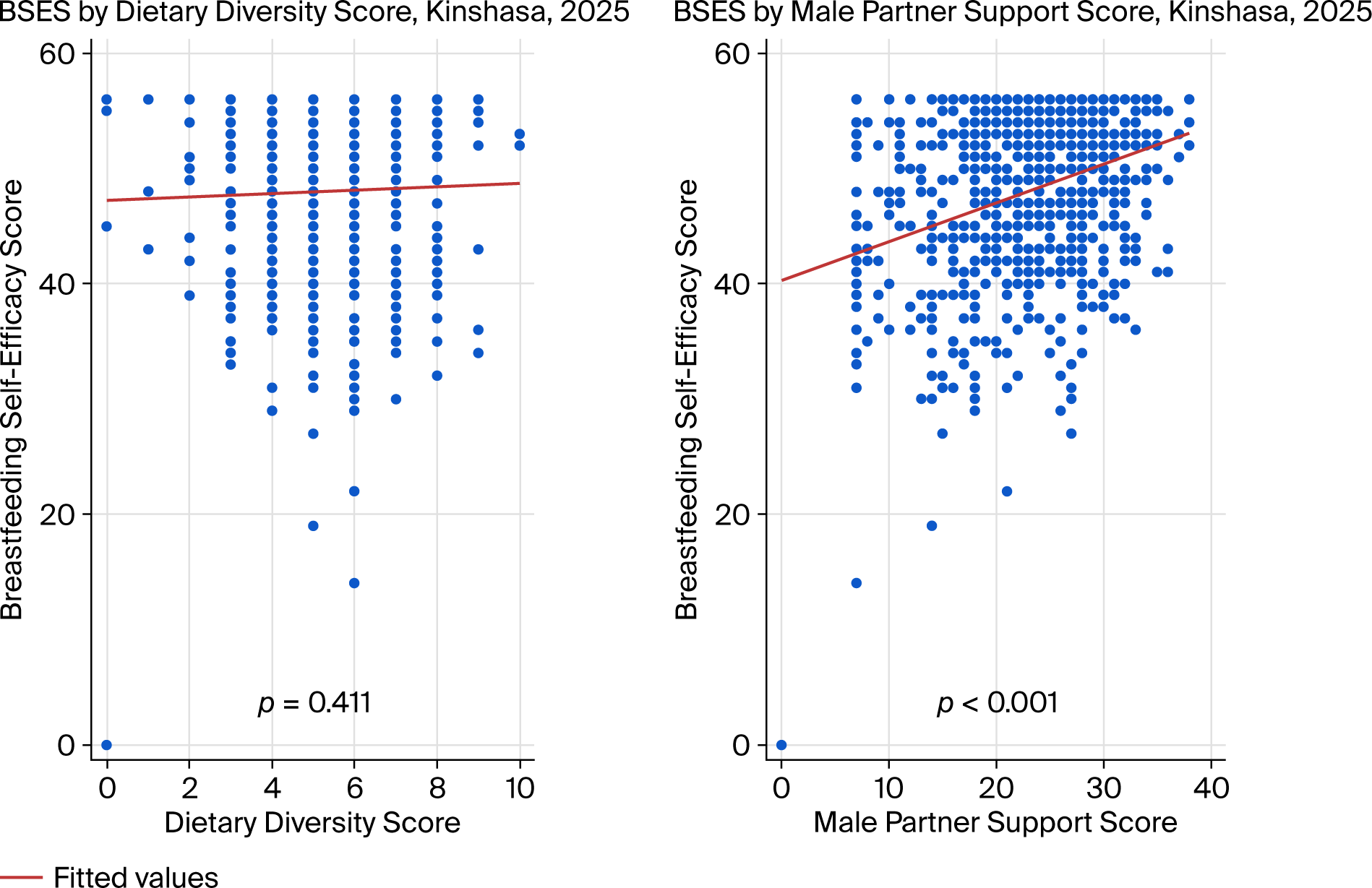

Supplement: Supplementary file 1 [file nutrients-18-00065-s001.zip › Figure-S2.png]

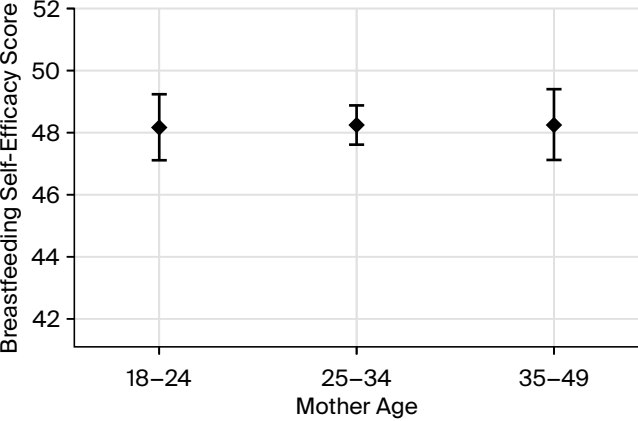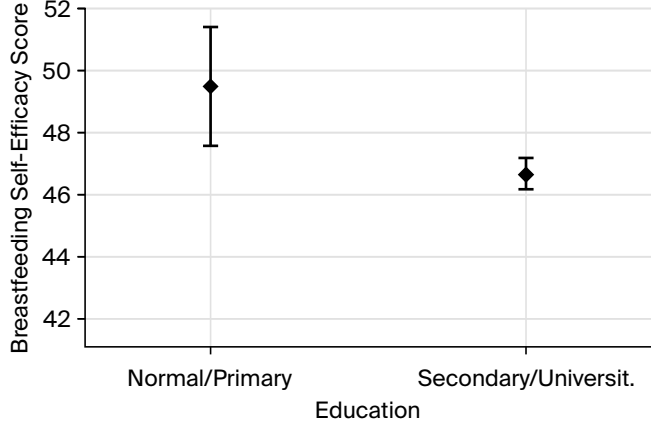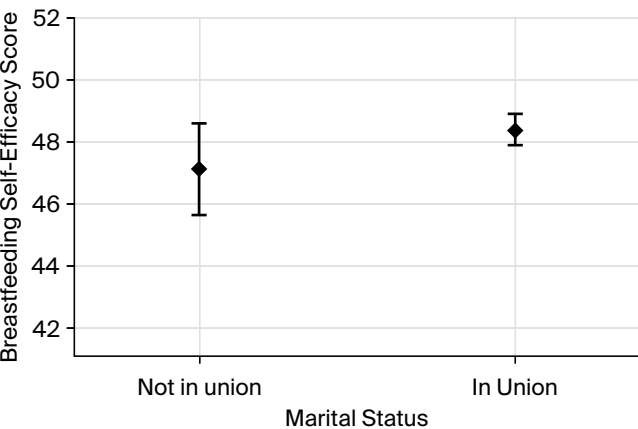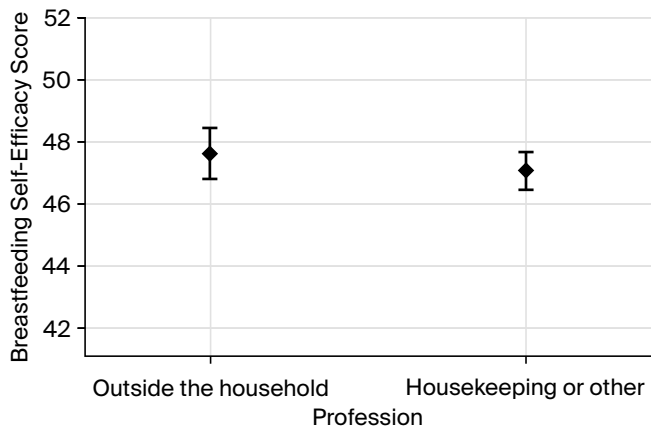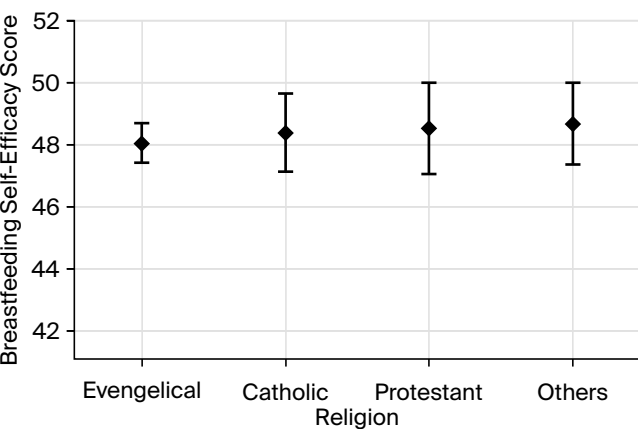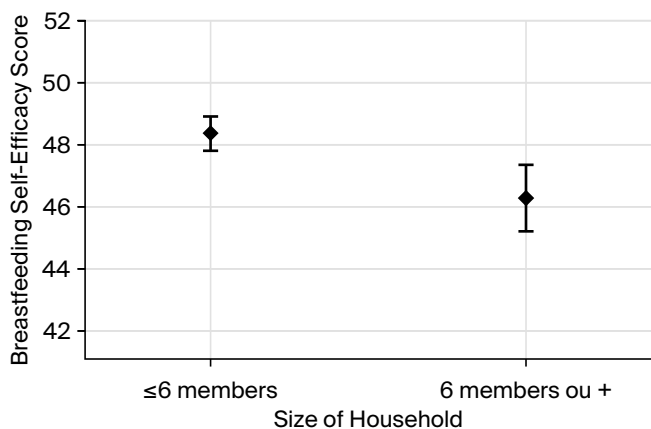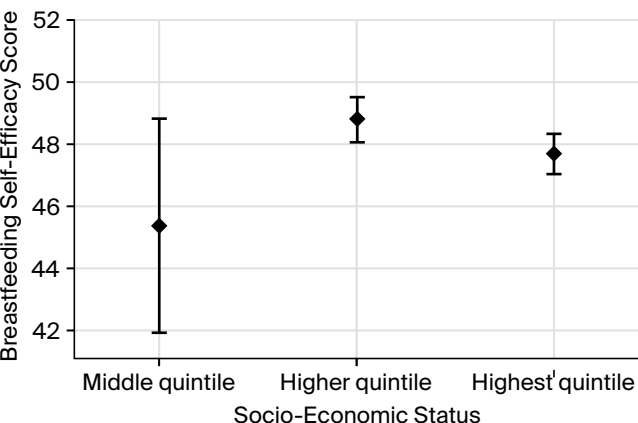

Supplement: Supplementary file 1 [file nutrients-18-00065-s001.zip › Figure-S3.pdf]

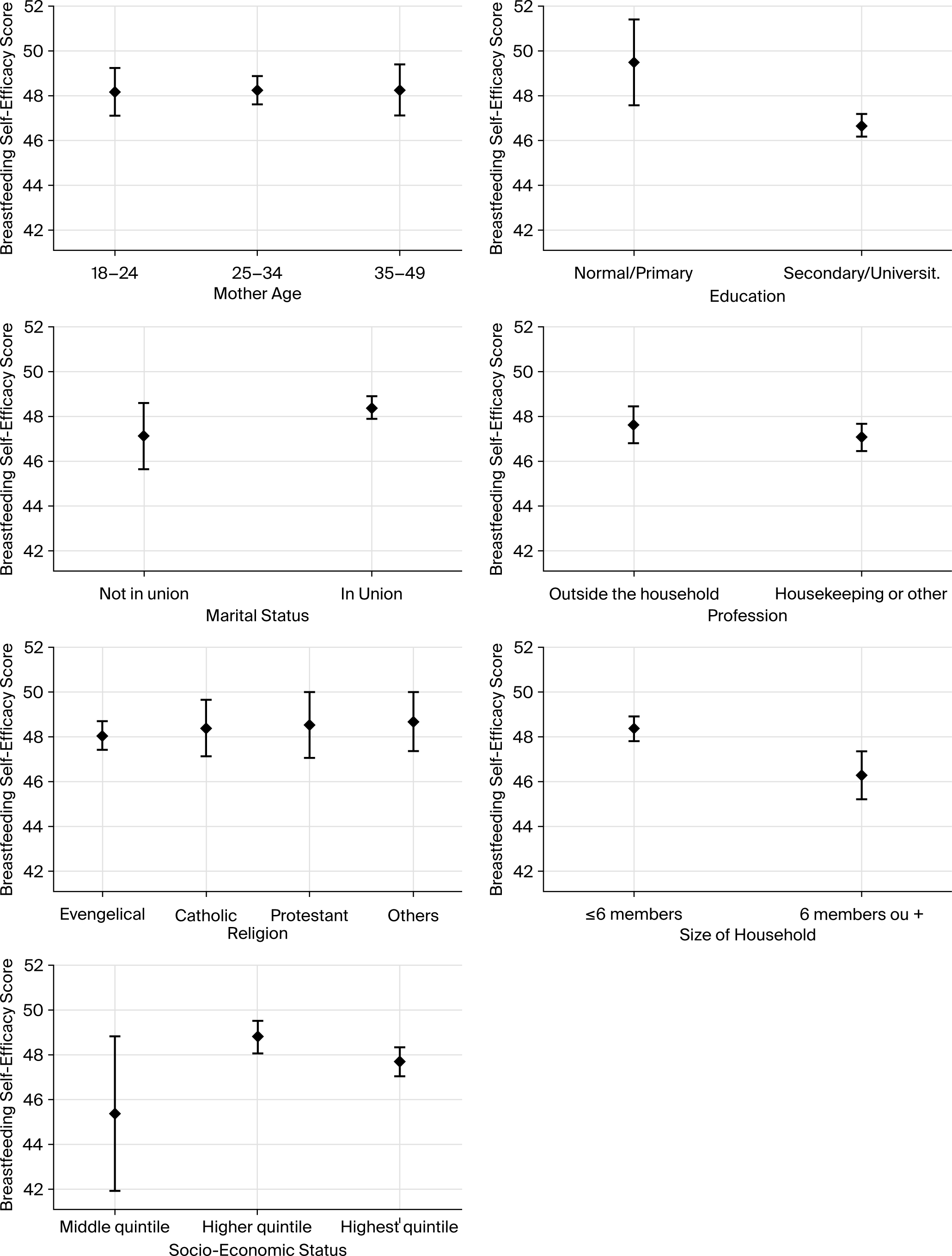

Supplement: Supplementary file 1 [file nutrients-18-00065-s001.zip › Figure-S3.png]

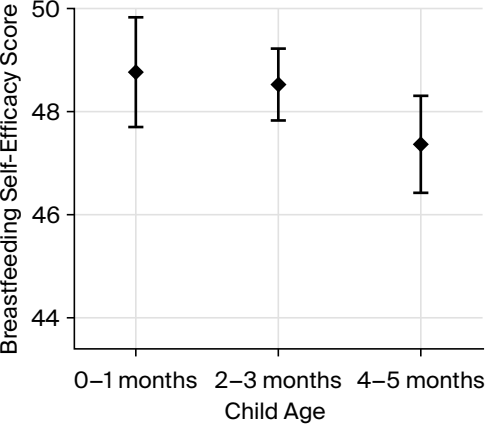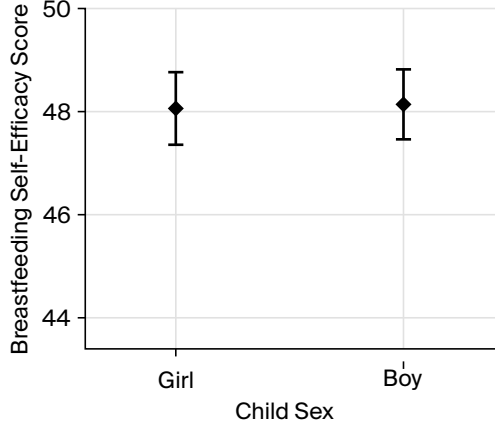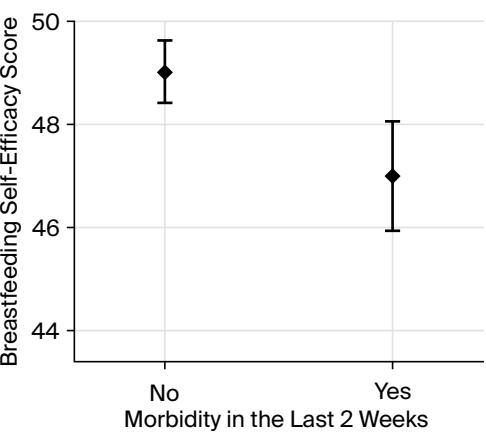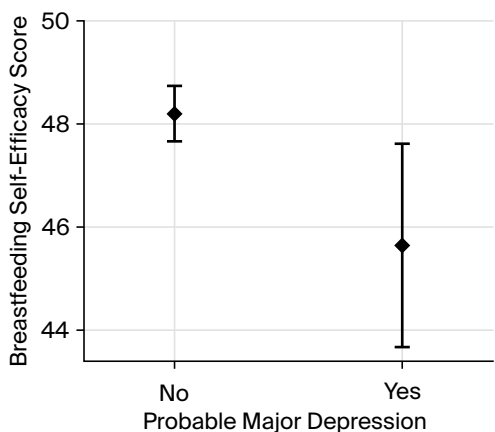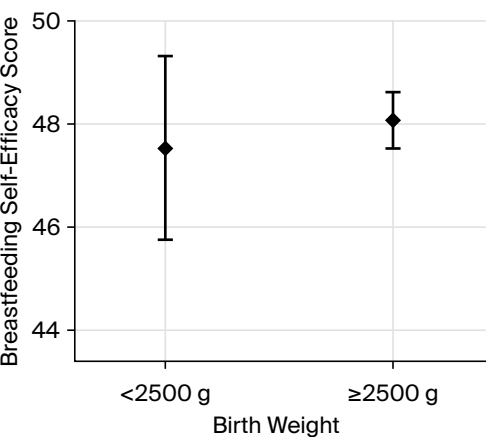

Supplement: Supplementary file 1 [file nutrients-18-00065-s001.zip › Figure-S4.pdf]

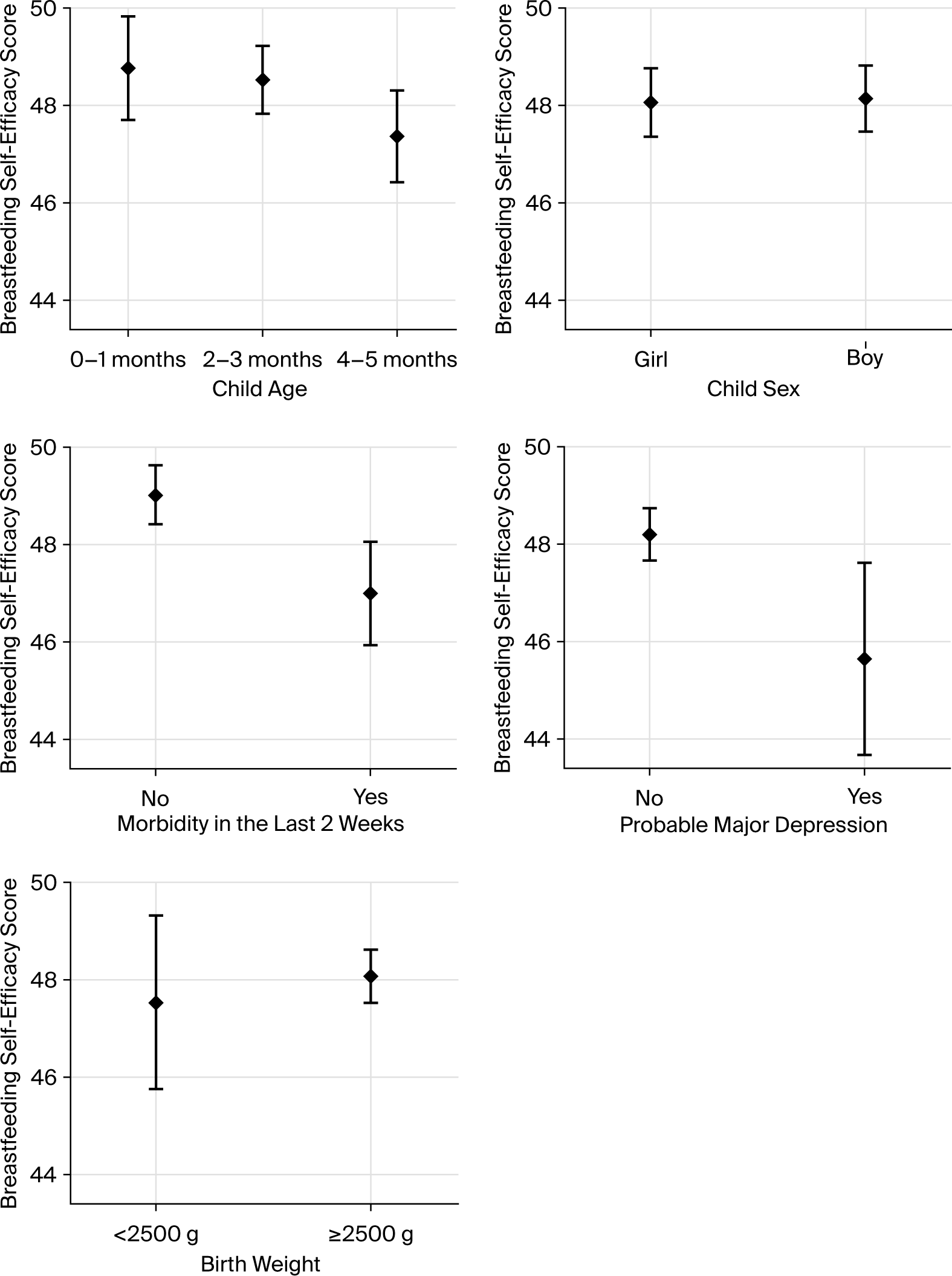

Supplement: Supplementary file 1 [file nutrients-18-00065-s001.zip › Figure-S4.png]

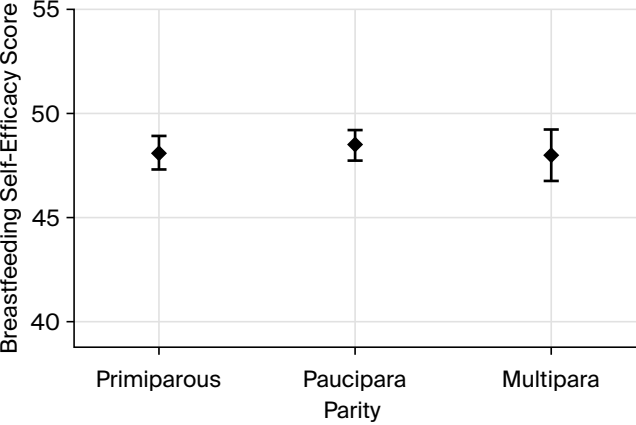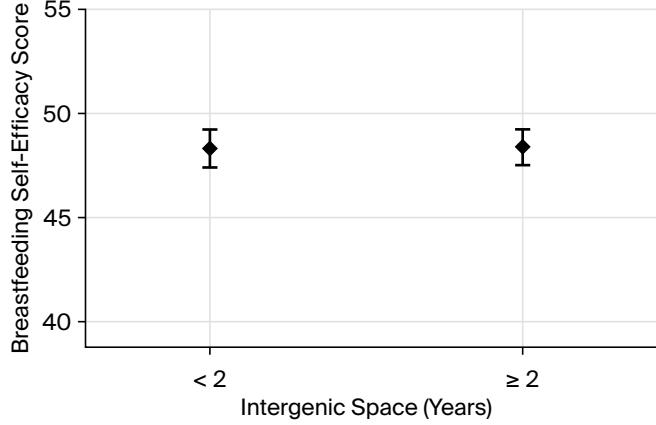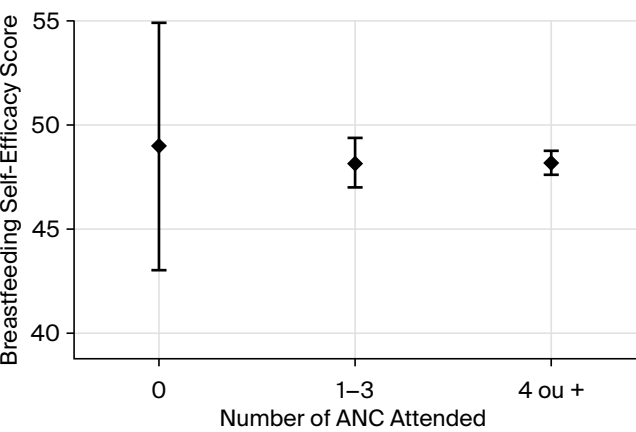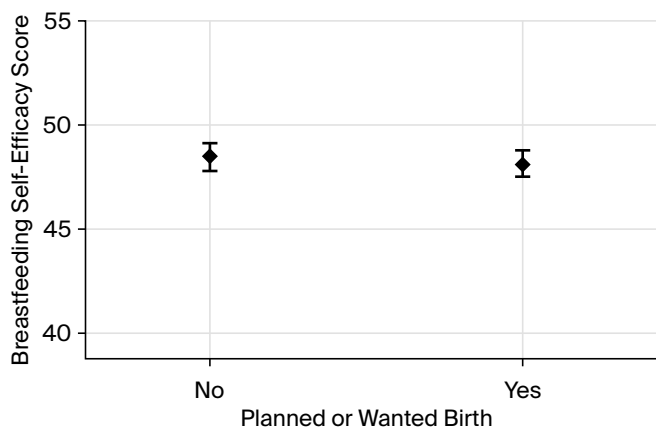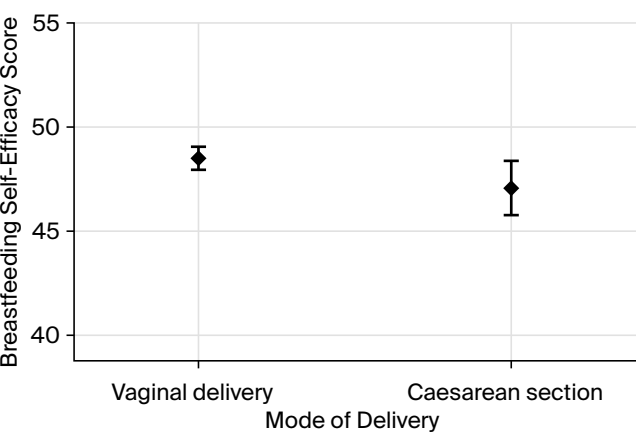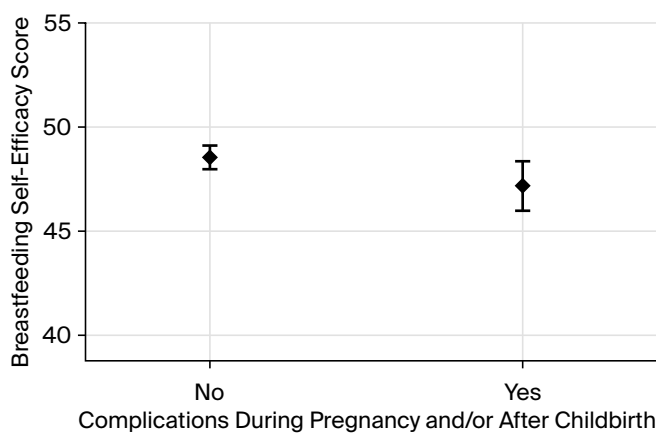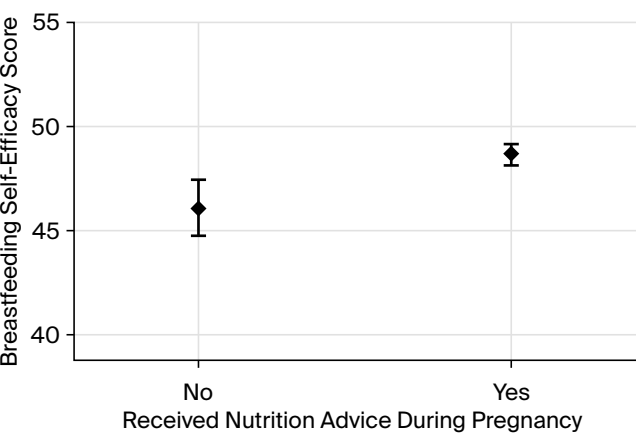

Supplement: Supplementary file 1 [file nutrients-18-00065-s001.zip › Figure-S5.pdf]

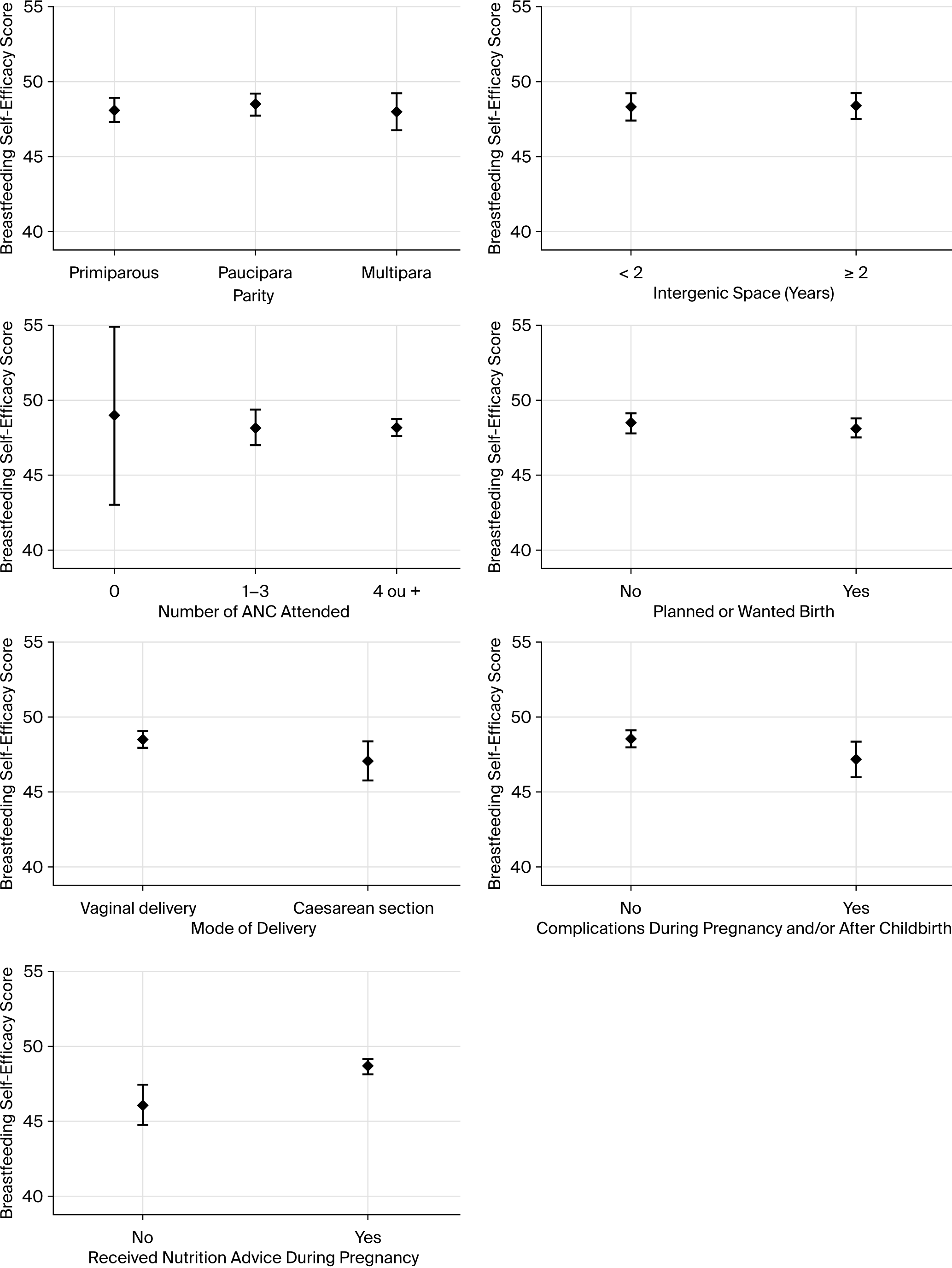

Supplement: Supplementary file 1 [file nutrients-18-00065-s001.zip › Figure-S5.png]
